# Mercerization Optimization of Bamboo (Bambusa vulgaris) Fiber-Reinforced Epoxy Composite Structures Using a Box–Behnken Design

**DOI:** 10.3390/polym12061367

**Published:** 2020-06-17

**Authors:** Mohamad Zaki Hassan, Siti Amni Roslan, S. M. Sapuan, Zainudin A. Rasid, Ariff Farhan Mohd Nor, Mohd Yusof Md Daud, Rozzeta Dolah, Mohd Zuhri Mohamed Yusoff

**Affiliations:** 1 Razak Faculty of Technology and Informatics, Universiti Teknologi Malaysia, Kuala Lumpur, Jalan Sultan Yahya Petra, Kuala Lumpur 54100, Malaysia; yusof.kl@utm.my (M.Y.M.D.); rozzeta.kl@utm.my (R.D.); 2 Malaysia-Japan International Institute of Technology, Universiti Teknologi Malaysia, Kuala Lumpur, Jalan Sultan Yahya Petra, Kuala Lumpur 54100, Malaysia; amnihusna90@gmail.com (S.A.R.); arzainudin.kl@utm.my (Z.A.R.); ariffmdnor@yahoo.com (A.F.M.N.); 3 Advanced Engineering Materials and Composites Research Centre, Department of Mechanical and Manufacturing Engineering, Universiti Putra Malaysia, Serdang 43400 UPM, Malaysia; sapuan@upm.edu.my (S.M.S.); zuhri@upm.edu.my (M.Z.M.Y.); 4 Laboratory of Biocomposite Technology, Institute of Tropical Forestry and Forest Products (INTROP) Universiti Putra Malaysia, Serdang 43400 UPM, Malaysia

**Keywords:** alkaline treatment, optimization, bamboo fibers, response surface methodology

## Abstract

The objective of this research is to optimize the alkaline treatment variables, including sodium hydroxide (NaOH) concentration, soaking, and drying time, that influence the mechanical behavior of bamboo fiber-reinforced epoxy composites. In this study, a Box–Behnken design (BBD) of the response surface methodology (RSM) was employed to design an experiment to investigate the mercerization effect of bamboo fiber-reinforced epoxy composites. The evaluation of predicted tensile strength as a variable parameter of bamboo fiber (Bambusa vulgaris) reinforced epoxy composite structures was determined using analysis of variance (ANOVA) of the quadratic model. In this study, a total of 17 experiment runs were measured and a significant regression for the coefficient between the variables was obtained. Further, the triangular and square core structures made of treated and untreated bamboo fiber-reinforced epoxy composites were tested under compressive loading. It was found that the optimum mercerization condition lies at 5.81 wt.% of the NaOH, after a soaking time of 3.99 h and a drying time of 72 h. This optimum alkaline treatment once again had a great effect on the structures whereby all the treated composite cores with square and triangular structures impressively outperformed the untreated bamboo structures. The treated triangular core of bamboo reinforced composites gave an outstanding performance compared to the treated and untreated square core composite structures for compressive loading and specific energy absorbing capability.

## 1. Introduction

Natural fiber-reinforced composites have been utilized as reinforcements to replace glass and carbon fiber-reinforced composites for various applications, ranging from sports equipment to advanced aerospace structures, due to their unique characteristics, including biodegradability, high specific strength-to-weight ratio, good acoustic resistance, good sustainability, good abrasion resistance, and outstanding energy absorption [1,2,3]. Many attempts have been made in order to improve the properties of composites including the use of natural fiber as a single fibrous nanocomposite for filling in the polymer matrix. Enayati et al. [4] successfully used an electrospinning technique to disperse a wheat straw cellulose nanofiber in polyvinyl alcohol. This matrix modification approach has proved that the Young’s modulus of the composite was significantly increased by over six times that of the neat polymer [5].

Natural fiber-based composites are mainly limited to non-structural applications by the incompatible behavior between the natural fiber and the synthetic polymer matrix due to a hydrophobic interaction with the hydrophilic cellulose. In order to combat this issue, the fiber surface of a natural fiber can be modified using chemical treatment. Alkalization, acetylation, benzoylation, bleaching, and silane treatment are frequently used for chemical surface modification of the natural fiber surface. Alkaline treatment, known as the mercerization technique, involves the immersion of natural fibers in concentrated sodium hydroxide (NaOH) solution and has been considered as one of the most popular and cost-effective methods. Here, NaOH reacts with the hydroxyl group of the natural fibers to remove the cementing materials, including hemicellulose, lignin, wax, and oils, that surround the external surface of the fiber leading to an increase in the surface roughness and fiber-matrix bonding interactions.

Many studies have proven that the chemical modification of natural fibers can increase the mechanical properties of the composite, fiber-matrix adhesion, and compatibility of the natural fiber. Several types of natural fibers, such as kenaf [6], banana [7], and sugar palm [8], were treated using this method and improved the thermal, impact, and barrier properties of fibers in various thermoset and thermoplastic matrices. Khan et al. [9] studied the effect of NaOH concentration on bamboo fiber-reinforced epoxy composites examining tensile strength and Mode I fracture behavior. They found that treatment of bamboo fibers with 6 wt.% concentration NaOH offers the highest ultimate tensile strength and maximum Mode-I plane strain fracture toughness (K_IC_) of the composite laminate. In addition, Cai et al. [10] immersed abaca fibers in 5, 10, and 15 wt.% NaOH solutions for 2 h, and evaluated the effects of the alkali treatments on the mechanical characteristics and interfacial adhesion of the abaca fiber/epoxy composite. They suggested that 5 wt.% NaOH treatment of abaca fibers increases their crystallinity, tensile strength and Young’s modulus compared to untreated fibers, as well as improving the fiber/epoxy interfacial shear strength. The effects of alkali treatment and elevated temperature on the mechanical properties of bamboo fiber-reinforced composites were conducted by Manalo et al. [11]. The bamboo fibers were treated with between 4 and 8 wt.% NaOH and then randomly oriented in a polyester matrix. Here, an optimum NaOH concentration of 6 wt.% was suggested to give the highest mechanical properties. In contrast, Rao et al. [12] suggested that 1% concentration of NaOH is the optimum condition to treat a bamboo polymeric composite. On the other hand, Nirmal et al. [13] claimed that a 6 wt.% NaOH concentration is best for treating natural fiber and capable of removing excess moisture thoroughly whilst exhibiting excellent interfacial adhesion strength. However, evidence that the NaOH concentration alone is the factor that affects the bonding between a matrix and a natural fiber is still inconsistent.

Recently, researchers have shown an increased interest in determination of the optimum condition for alkaline treatment of the natural fiber by using optimization tools. Vardhini et al. [14] optimized the lignin removal from banana fiber with different concentrations of NaOH, time and temperature at three levels using Box–Behnken experimental design. The optimum conditions for lignin decomposition are identified as alkali concentration 11 g/L, treatment time of 150 min, and temperature at 90 °C. Response surface methodology (RSM) was also employed by Kim et al. [15] to optimize alkaline pre-treatment conditions for maximum glucose yield from rice straw with respect to NaOH concentration (1.0–4.0%), reaction temperature (60–100 °C), and pre-treatment time (30–90 min). The maximum glucose yield of 254.5 ± 1.2 g kg^−1^ biomass was obtained at the optimum treatment condition of 2.96%, 81.79 °C, and 56.66 min. The ANOVA test revealed that the model and all independent parameters were considered statistically significant at 95% confidence level. Furthermore, Singh et al. [16] used a three-level Box–Behnken design (BBD) method to optimize the mass fraction of fibers, the percentage of crosslinking and the plasticizing agent. Initially, they treated the sugarcane bagasse fiber with 6 wt.% sodium hydroxide. The model predicts that the optimum condition for maximum tensile strength is archived with a matrix content of 11% glyoxal, 17.7% glycerol, and 51.3% soy protein isolate by weight.

In this study, the variables that usually impact on the chemical treatment of bamboo, such as concentration of NaOH, soaking time and drying period are selected. To obtain the optimum values for these parameters, a study using a Box–Behnken design (BBD) for the response surface methodology was employed. The tensile behavior is determined as a function of NaOH concentration, soaking time and drying period; then a quadratic function equation is proposed. Further, the compression behavior of triangular and square bamboo/epoxy composite core structure were evaluated.

## 2. Materials and Methods

Initially, BBD was used to develop an experimental design for the alkaline treatment conditions for bamboo fibers. The treatment conditions consist of three selected variables: including sodium hydroxide (NaOH) concentration, soaking, and drying time. The design points for each variable in combinations of the high, middle, and low factor levels are derived from Design-Expert (6.0.11) software (Stat-Ease Inc., Minneapolis, MN, USA).

### 2.1. Design of the Experiment

The selection of factor levels for the NaOH concentration (X_1_), soaking period (X_2_), and time for bamboo to dry (X_3_) are described as follows:

3 wt.% ≤ *NaOH concentration* (X_1_) ≤ 9 wt.%

3 h ≤ *soaking time* (X_2_) ≤ 9 h

24 h ≤ *drying time* (X_3_) ≤ 72 h

Table 1 tabulates the coded and actual levels of the determining variables in this study. The relationship between the coded values and actual values is described in Equation (1).

(1) $$x_{i}=\frac{X_{i}-X_{o}}{\Delta X_{i}}\;i\;=\;1,2,3$$

where *x_i_* and *X_i_* are the coded and actual values of the independent variable, respectively. *X_o_* is the actual value of the independent variable at the middle level, and Δ*X_i_* is the step change of *X_i_*. Then, the input data of Table 1 are randomized and modelled by BBD.

Table 2 lists the design of the experiment determined by the BBD. This model calculated a total of 17 experimental runs consisting of 12 distinct runs and five replications.

### 2.2. Experimental Method

**Figure 1:**
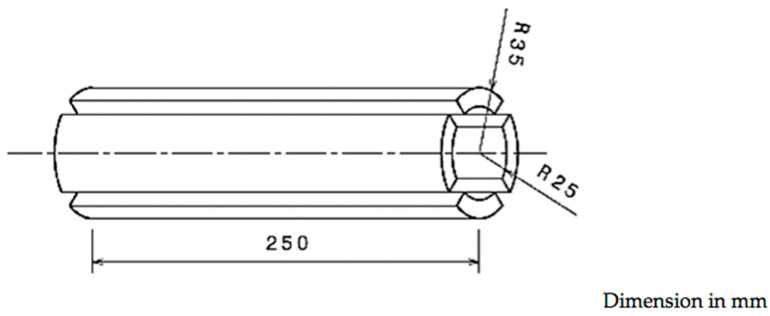
Schematic diagram of the bamboo culm cutting process.

The bamboo used in this study is Bambusa vulgaris which is generally found in well-drained soil in many tropical regions. Here, the Bambusa vulgaris bamboo was collected from Jeli-Kelantan, on the East Coast of Malaysia and was taken out at approximately 5 m above the ground. In order to fabricate the tensile specimen, the bamboo culm was cut into several smaller pieces using a handsaw and each piece was sliced into four parts as illustrated in Figure 1. Further, these parts were then dried under the sun for a week.

#### 2.2.1. Alkaline Treatment and Bamboo Fiber-Reinforced Epoxy

**Figure 2:**
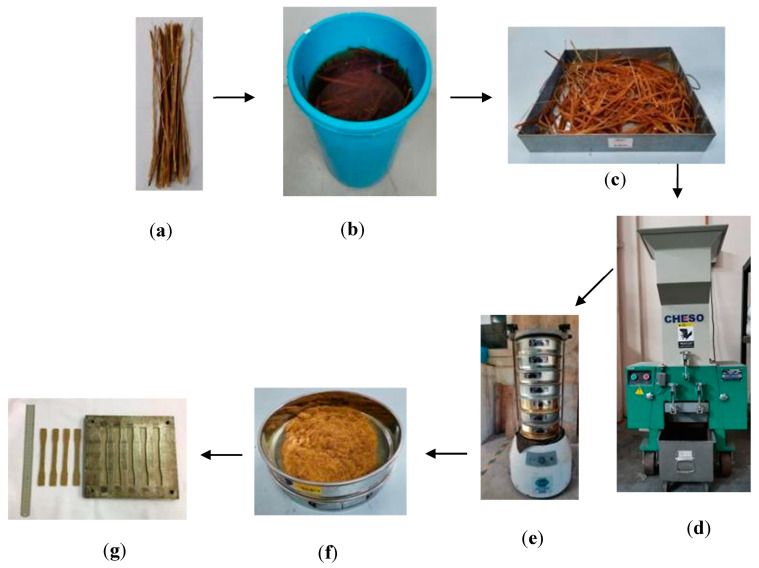
Typical chemical treatment process and dumbbell-shaped bamboo epoxy composite fabrication where (**a**) raw bamboo fibers, (**b**) soaking fibers into NaOH solution, (**c**) dried bamboo fibers, (**d**) grounding down fibers, (**e**) sieved the fibers, (**f**) obtaining the desired short bamboo fibers and (**g**) tensile-molded specimens.

Initially, the bamboo culm was sliced using a sharp straight-edged knife, giving a longitudinal thin bamboo as shown in Figure 2a. To eliminate any dirt and surface impurities, the sliced bamboo was washed with deionized water, before being soaked in 3, 6, and 9 wt.% [17] concentration NaOH (Figure 2b). These bamboos were again washed using distilled water for 3, 6 and 9 h [18] according to the conditions suggested by the Box–Behnken design, and then left to dry for 24, 48, and 72 h in a circulating oven at 80 °C [19]. The dried bamboo, as shown in Figure 2c, was then ground down using a Cheso Model N3 crusher machine (Figure 2d). In order to maintain the fiber length, a multi stage sieve fixed to a rotational shaker model BS410/1986 (Endecots Ltd., London, England) (Figure 2e) was used. Figure 2f shows the short fiber was obtained from this shaker when operated at 275 rpm for 45 min. Then, the epoxy-resin (EpoxAmite™, Smooth-On Inc., Pennsylvania, PA, USA) matrix with fibers was gradually mixed with fiber at 29.86 (wt.%) of fiber loading [20]. Figure 2g illustrates the dumbbell-shaped specimens adhered to the ASTM D638 standard [21] that were cured overnight using a rigid steel mold. These flat specimens were typically dimensioned as 165 mm (overall length), 13 mm (width) × 57 mm (gauge length) × 3 mm (thickness) with an average weight of 9.50 ± 0.55 g.

#### 2.2.2. Tensile Test of Bamboo Fiber-Reinforced Epoxy

Tensile testing was carried out to investigate the effect of alkaline treatment on the tensile properties of the bamboo-reinforced epoxy composite. A specimen was mounted on a Shimadzu universal testing machine (Shimadzu Corporation, Kyoto, Japan) and the test was conducted at a crosshead displacement rate of 1 mm/min using a 10 kN load cell. The load displacement relationship was recorded and the test was stopped upon specimen fracture. The mechanical properties including tensile stress, σ (MPA), strain-at-break, *ε* (%), and Young’s modulus *E* (GPa) of the composite, were deduced from the stress–strain curves. At least five specimens were tested for each condition and the average value subsequently recorded.

#### 2.2.3. Compression Test of Bamboo Fiber-Reinforced Epoxy Structure

**Figure 3:**
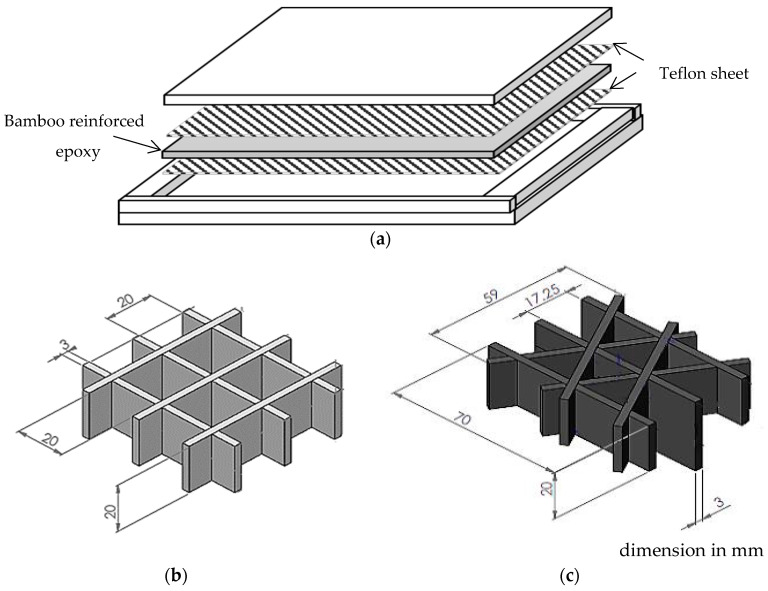
Schematic of (**a**) the bamboo-reinforced epoxy panel, with the (**b**) the square and (**c**) the triangular core structures.

The compression test was conducted to investigate the compressive behavior and energy absorption characteristics of triangular and square core structures made of bamboo composites. Initially, bamboo fibers were gradually mixed with epoxy-resin paste and placing in picture frame mold. In order to avoid the composite stacking to the mold, thin layers of Teflon sheet were used as shown in Figure 3a. Further, the panel was then heated to 70 °C for one hour under a pressure of 1 bar using a hot press machine before leaving them to cure. Figure 3b,c shows the construction of triangular and square core structures, respectively. The design of these structures were adopted from Zuhri et al. [22]. The core structures were prepared by cutting the composite into strip sized 70 mm × 20 mm with a series of 10 mm depth slots at a distance of 20 mm apart on each strip using a laser cutter. These triangular and square cores were assembled using a slotting technique giving a total height of 20 mm.

The compression tests for both core structures were carried out using Shimadzu universal testing machine according to ASTM C365 [23]. The honeycomb core structure was then placed between a rigid platen and the base and loaded at a crosshead displacement rate of 1 mm/min. The tests were stopped when the specimens were totally crushed. The test was repeated three times for each condition.

## 3. Results and Discussion

### 3.1. Tensile Properties of Bamboo Reinforced Epoxy Composites

The tensile properties of bamboo composite with different treatment conditions are tabulated in Table 3. The treated bamboo at the median NaOH concentration, the longest soaking time and dry duration shows the highest tensile strength value (labelled as specimen run 7), whereas the lowest tensile strength was specimen run 2. By comparing both results, the bamboo composite treated with 6 wt.% NaOH, with 3 h soaking and 72 h drying time, exhibited the highest tensile strength, which is 28% higher than the 9 wt.% NaOH—6 h soaking—24 h drying time sample. This result shows that 6 wt.% NaOH, with 3 h soaking and 72 h drying time, could increase the tensile strength significantly compared to the other treatment conditions. It can be suggested that a bamboo-reinforced epoxy composite treated under these conditions has had the impurities effectively removed from the bamboo surface and so offers a better fiber–matrix adhesion along with the interfacial loading transfer between the fiber and the matrix [18]. The results also show that there is an increase in tensile strength value for the longer drying time of the composite. This reason for this longer drying time is to ensure the removal of the NaOH residue through the fiber surface [24]. El Sheikh et al. [25] mentioned that residual NaOH has a negative effect on the fiber-matrix adhesion, which explains the decreasing value of the mechanical properties of the structure. In addition, the tensile strength of the bamboo composites steadily increased with increased of the drying time. Zikri et al. [26] reported that the tensile strength of the fiber-epoxy composites significantly decreased by more than 50% under wet conditions. Here, under high moisture content, the water molecules within the composites weaken the intermolecular hydrogen bonding between the cellulose molecules of the fiber and the water molecules [27], hence the interfacial adhesion between the fiber and the matrix was significantly reduced.

As shown in Table 3, the tensile strength significantly decreased with increasing to 9 wt.% of NaOH concentration except at higher soaking and drying time. This reduction in mechanical behaviour was also reported by Misra et al. [28] when the sisal fibers were treated with 10 wt.% NaOH concentration. Moreover, Oushabi et al. [29] stated that, at high concentrations of NaOH, the fiber pull-out energy that correlated with the fracture energy decreases due to irregularities in the contact surfaces between the fiber and the matrix. This massive delignification process tends to reduce the diameter and excessively damage the fiber leading to poor fiber-matrix adhesion [29]. There are inconsistent trends for strain-at-break and Young’s modulus for all treatment conditions, but the differences between them are quite insignificant and can be considered negligible. However, the improvement up to 214.4% detected on the strain at break for alkaline treated bamboo compared to untreated one. The alkaline treatment made the composites more elastic than brittle untreated bamboo/epoxy composites.

**Figure 4:**
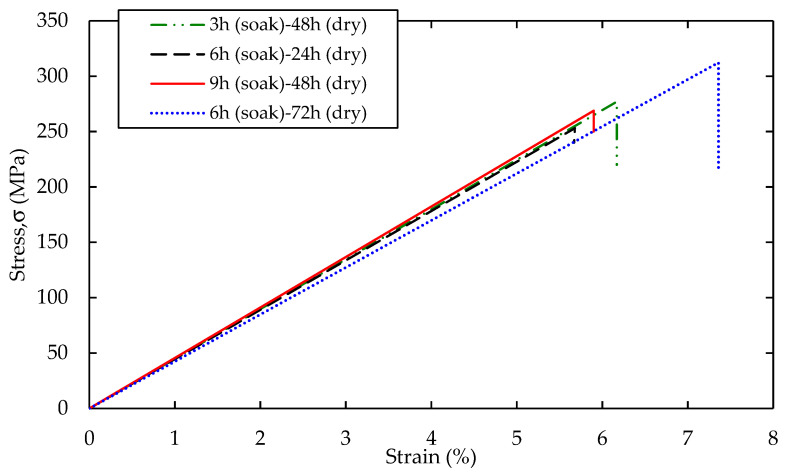
Typical stress-strain curves bamboo reinforced epoxy composites treated with of 3 wt.%, NaOH concentration.

Figure 4 shows the stress-strain curves for bamboo reinforced epoxy composites treated with 3 wt.%, concentrations at different soaking and drying time. The bamboo treated with 3 wt.% NaOH, 6 h soaking and 72 h drying, yields a higher tensile strength with a lower deformation compared to the bamboo fibers under treatment conditions of 3 h soaking with 48 h drying time, 6 h soaking with 24 h drying time, and 9 h soaking with 48 h drying time. The enhancement of 23.4% is revealed at median soaking time and higher of the drying time that compare with other conditions at same 3 wt.% NaOH concentration. Fracture of the specimen may be due to the formation of peaks and valleys that that concentrate the load at a particular point on the composites as a result of higher mechanical properties. Meanwhile, the relationship between soaking and drying time is discussed in details at BBD discussion section. Manalo et al. [11] conducted a tensile test of treated bamboo fiber polyester composites. They indicated the average tensile stress is 19 MPa for those specimens, which is 15 fold lower than reported here. Besides the different type of polymer matrix and matrix adhesion between the fiber–matrix reinforced composite, they also suggested that the tensile behavior of bamboo fibers is dominated by the orientation of the fiber.

### 3.2. Box Behnken Design (BBD) Analysis of Treated Bamboo Reinforced Epoxy Composites

By applying the analysis of variance, the quadratic model for tensile strength of the treated bamboo reinforced epoxy composite is expressed in Equation (2). The comparison between calculated tensile strength and the experimental findings for the treated bamboo reinforced epoxy composites are summarized in Table 4.

Tensile strength = 117.115 + 30.39(X_1_) + 16.10(X_2_) + 1.14(X_3_) − 2.92(X_1_^2^) − 1.16 (X_2_^2^) + 0.003 (X_3_^2^) − 0.27(X_1_ X_2_) + 0.066(X_1_ X_3_) − 0.073(X_2_ X_3_) (2)

where *X*_1_*, X*_2_, and *X*_3_ are corresponding coded variables of NaOH concentration (*X*_1_), soaking period (*X*_2_), and drying time (*X*_3_).

A good agreement between actual and predicted data occurs when the residual and the error for the actual tensile strength are less than 2%. Here, this empirical model fitting is used to validate the accuracy of iteration of the three chosen factors using *F*-test and regression coefficient, *R*^2^. Table 5 tabulates the statistical measurements from ANOVA. The measured values the coefficient of determination (*R*^2^) close to 1 indicated the degree of fit. Joglekar et al. [30] reported that a good model fit would yield an R^2^ value of at least 0.8. The mean of the response model calculated in this study explains the reaction of the tensile strength with the design factors very well with a value of Adj-R^2^ value of 0.986 and a Predicted-R^2^ of 0.924 at a 95% confident level. The signal-to-ratio was measured by a term called adequate precision. In this study, the ratio was 282.37, which is larger than four, and is thus considered acceptable [31]. Further, the coefficient of variation (C.V.) is less than 10% [32], which implies that the model is highly reliable.

The analysis of variance data for the tensile strength of the treated bamboo specimens are tabulated in Table 6. This result is used to identify the significance of the model and its parameters following Fisher’s F-test and the Student *t*-test. As can be seen from Table 6, larger F-values (F_model_ = 129.17) and a smaller *p*-value (*p* < 0.0001) show the high significance of the coefficient level. A *p*-value less than 0.05 indicates that the model is statically significant, while the value larger than 0.10 indicates that the model is not significant [33]. In addition, the correlation coefficient of quadratic terms of NaOH concentration and drying time are implied to be highly significant. This means that these two factors influence the removal of the hemicellulose content of the fibers. Sari et al. [34] reported that alkaline treatment decreased the moisture content of the fiber, whereas the interstices between the groups of microfibrils would be blocked, thereby reducing water accessibility. In addition, a longer drying time may suggest an additional interfacial bonding strength interaction of the polymer matrix when bamboo is used as the fiber-reinforced in a composite laminate.

### 3.3. Diagnostic Plots of the Composites

**Figure 5:**
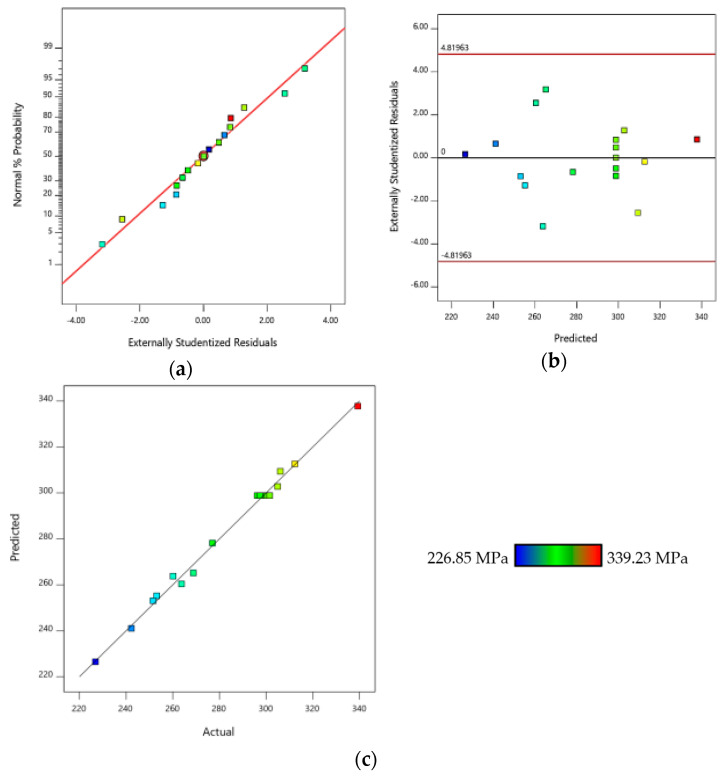
Diagnostic plots values of (**a**) normal probability versus residuals, (**b**) residuals versus predicted and (**c**) predicted versus actual of tensile strength bamboo reinforced epoxy composites.

Figure 5 presents the diagnostic plots of tensile strength for the treated bamboo reinforced epoxy composites. It is evident from these figures that all plots have fulfilled the general statistical assumptions about residuals by displaying a normal data distribution. Figure 5a shows a typical normal probability distribution versus residuals. In the figure where the residuals fall near to the ideal line of regression there is good agreement with the normal distribution which suggests that the model fits the data satisfactorily [35]. On the other hand, Figure 5b presents the plot of residuals versus predicted values which show that the data points are randomly scattered across the plot without exceeding the upper and lower limit lines. In general, the studentized residuals should lie between +3 and −3 [36]. In Figure 5b, the optimization value was close to ±3, thereby, along with the pattern of the plot, confirming the accuracy of the proposed model. Figure 5c presents the predicted and actual values of response which seem to be in a reasonable agreement as the data points are well distributed around the reference line. Therefore, the distribution indicates the high accuracy of the model. To sum up, the proposed model is accurate and fits the data adequately [37].

**Figure 6:**
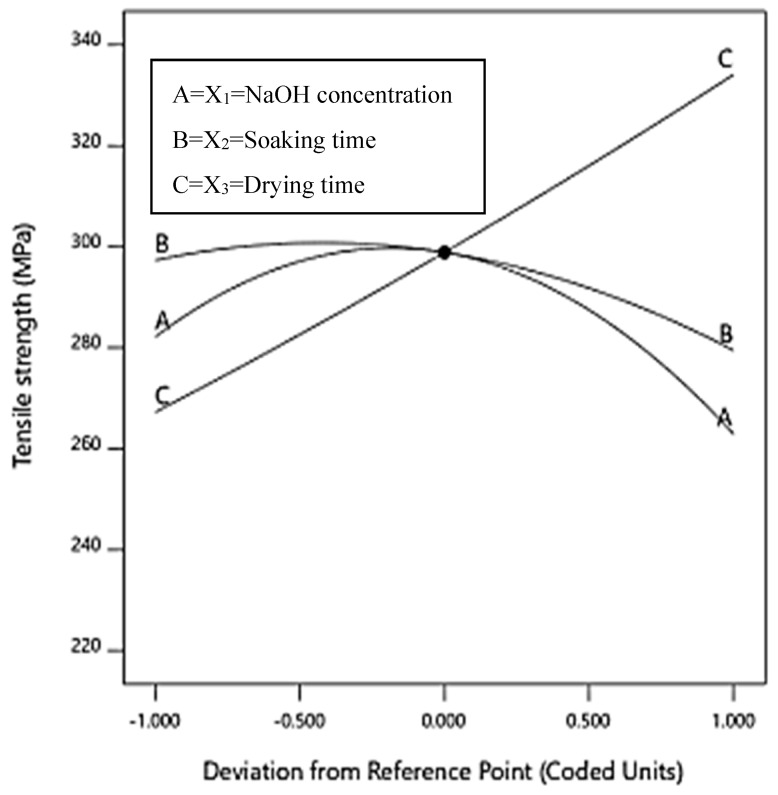
Perturbation plot showing the effect of all the variables on the tensile strength.

Figure 6 shows a perturbation plot which illustrates the effect of treatment conditions on the tensile strength of the treated bamboo reinforced epoxy composites. The plot also describes the changes of response as each variable moves from the centre point of the design space (*X*_1_ = 6.00, *X*_2_ = 6.00, *X*_3_ = 48.00). Here, it shows that the increase in drying time (*X*_3_) has an apparently positive effect on the response. In contrast, NaOH concentration (*X*_1_) and soaking time (*X*_2_) has an adverse effect on the response where the tensile strength decreasing with furthur increasing NaOH concentration and drying time after it treshold values, implying the decline of alkali effect. In this study, the value the determination indicates the reliability of the model [38].

### 3.4. Response Surface Plots

**Figure 7:**
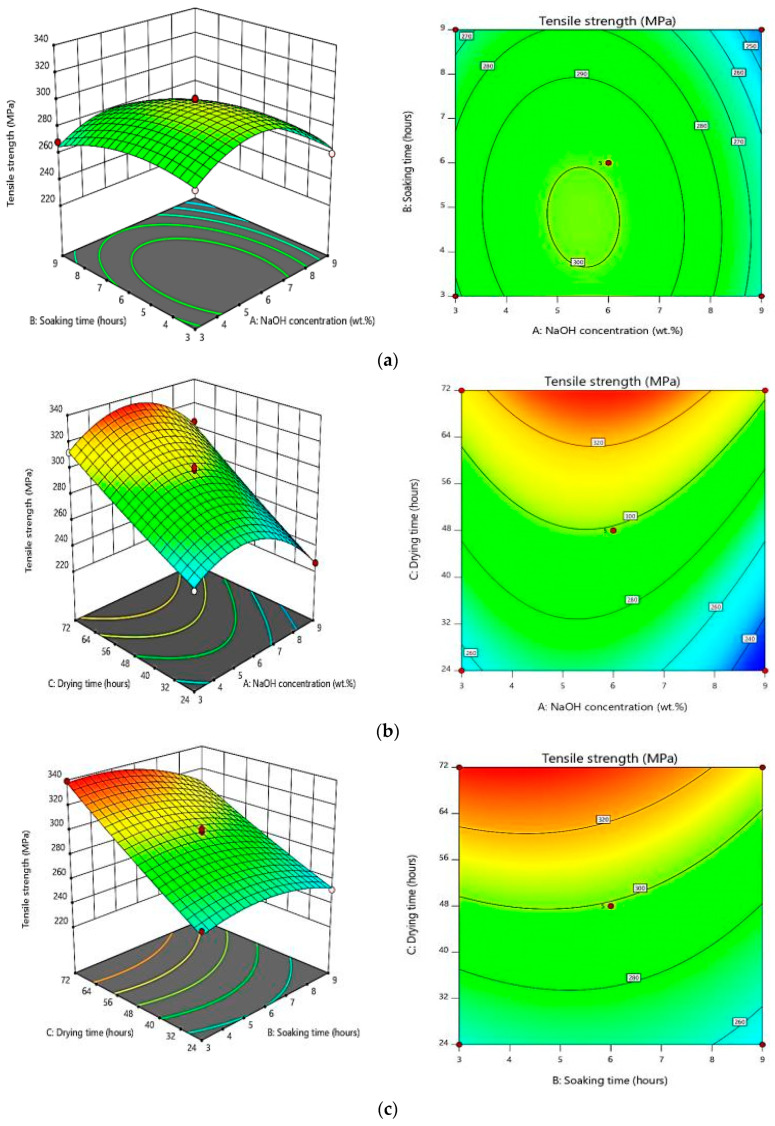
Typical 3D and 2D plots for the effect of (**a**) concentration versus soaking time (**b**) concentration versus drying time and (**c**) soaking time versus drying time on tensile strengths.

Figure 7a presents the response surface and corresponding contour plots for the interaction effect between NaOH concentration (*X*_1_) and soaking time (*X*_2_) on the tensile strength of bamboo-reinforced epoxy composites at 48 h drying time (*X*_3_). From the plots, the highest tensile strength of 298.89 MPa was recorded at the median NaOH concentration (6 wt.%) and the soaking time (6 h). In contrast, the tensile strength of the composites was the lowest (242.25 MPa) when treated with 9 wt.% concentration of NaOH for 9 h. This common shape of the 2D and 3D contour plots corresponds to the nature and extent of the relationship between factors [39].

Figure 7b presents the response surface and corresponding contour plots for the interaction effect of NaOH concentration (*X*_1_) and drying time (*X*_3_) on the tensile strength of bamboo-reinforced epoxy composites defaults at 6 h soaking time (*X*_2_). From the figures, the highest tensile strength of bamboo reinforced epoxy composites was recorded at 312.29 MPa after treatment of 3 wt.% concentration NaOH and drying for more than 60 h at room temperature. However, a drop of tensile strength value to 226.85 MPa is observed at the maximum treatment NaOH concentration (9 wt.% of NaOH) with minimum drying time (24 h). The deterioration occurring due to the alkali treatment had the greatest influence on the tensile strength as shown in Figure 6. The median range NaOH concentration delivers higher strength, however, both the lowest and the highest concentration contribute to the deterioration as the highest concentration reveals a more severe drop compared to the lowest one. This response surface is visualized regarding the tendency of each factor generated by the model surface using the second order polynomial (Equation (2)) to influence the tensile strength efficiency. The negligible effects are shown as a circular contour plot, while an elliptical contour plot indicates a prominent interaction [40].

On the other hand, Figure 7c shows the response surface and corresponding contour plots for the interaction effect of soaking time (*X*_2_) and drying time (*X*_3_) on the response in which the bamboo was treated with 6 wt.% concentration of NaOH. It can be seen that the highest tensile strength of bamboo reinforced epoxy composites was achieved at 339.27 MPa under the treatment condition of 6 wt.% NaOH with 3 h soaking and 72 h drying. The minimum tensile strength of bamboo reinforced epoxy composites when treated with 6 wt.% concentration of NaOH was reported to be 251.55 MPa after 9 h of soaking and drying for 24 h. In order to achieve a maximum tensile strength, Asim et al. [41] proposed that an optimal treatment condition is 6 wt. % concentration NaOH at 6 h of soaking time, which decreased the diameter of the pineapple leaf fibers.

### 3.5. Condition Optimization and Confirmation Tests

The tensile strength of bamboo-reinforced epoxy composites increased with the values of both NaOH concentration and soaking time up to their thresholds, before it then deteriorates as further increases occur. However, the drying time only produces a positive effect that tensile strength increase as the period increases. Following this, the tensile strength increases as the soaking time increased. Based on the analysis, it can be suggested that the highest tensile strength of the treated bamboo reinforced epoxy composite could be achieved up to 339.27 MPa under the treatment condition of 6 wt.% NaOH with 3 h soaking and 72 h drying time. Meanwhile, the minimum tensile strength was recorded at 226.85 MPa when the bamboo fiber was treated with 9 wt.% concentration of NaOH for 6 h and dried for 24 h.

**Figure 8:**
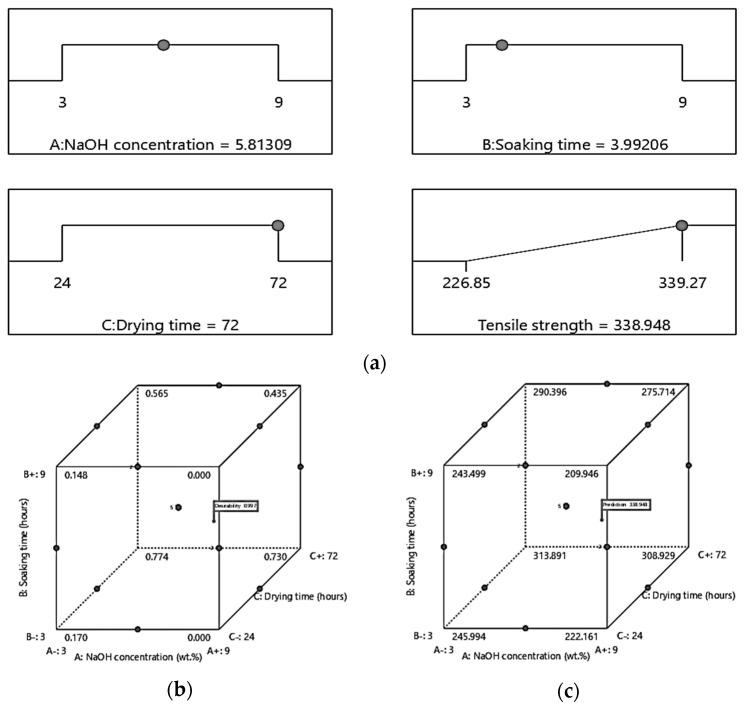
Typical (**a**) ramp, (**b**) desirability cube and (**c**) tensile strength cube represent the effect of NaOH concentration, soaking time and drying time under optimal conditions.

The optimization function in the Design Expert software was used to obtain the optimal conditions for the bamboo reinforced epoxy composite. The 3D contour plot obtained under optimal conditions is shown in Figure 8. According to the ramp plot shown in Figure 8a, the NaOH concentration of 5.81 wt.%, a soaking time of 3.99 h, and a drying time of 72 h are recorded. Here, the corresponding tensile strength is indicated at 338.27 MPa. Desirability values close to 1 were selected as the most effective parameters values with respect to the response factor. In this study, the desirability value was equivalent to 0.997 as shown in Figure 8b. The typical tensile strength cube for optimization is shown in Figure 8c. The good agreement between the experimental and predicted results verifies the validity of the model. To confirm the validity of the predicted optimal response, three additional experiments were carried out. For the three repeated experiment, the average tensile strength among was more than 85%. Wang et al. [42] examined the elementary aspect of the effect of alkali treatment on the cellulose of in bamboo fiber during the hydration reaction. This mercerization transition phenomenon leads to fibrillation by removing waxing materials. It also reduces the fiber diameter and increases its aspect ratio. In addition, Qian et al. [43] claimed that optimal alkali treatment increased the specific area of bamboo fiber, which could increase the possibility of matrix penetration into the fiber surface. These results are acceptable, indicating that a BBD is a very effective tool for optimizing individual factors in a new process.

### 3.6. Compression Properties of Bamboo Reinforced Epoxy Structures

**Figure 9:**
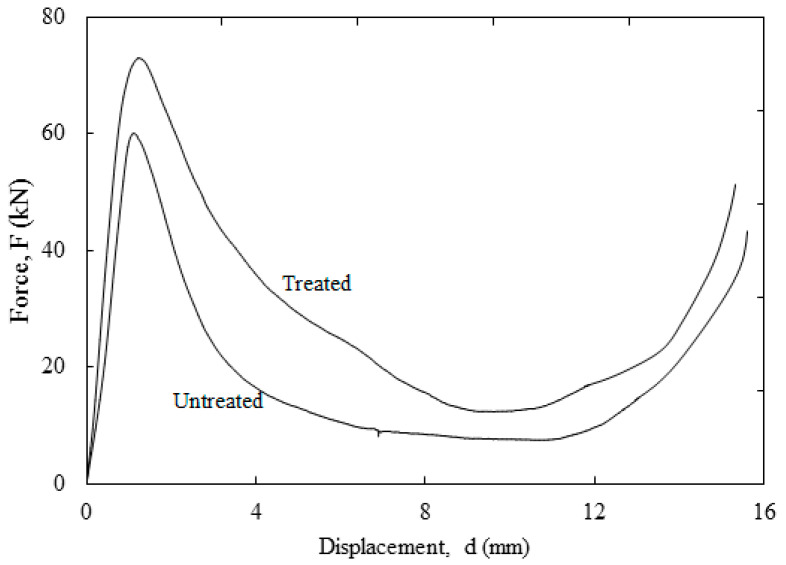
Typical force-displacement traces for untreated and treated square structures.

The compression tests of selected alkaline treatment conditions on triangular and square core structures made of bamboo reinforced epoxy composite were examined. Figure 9 shows the force-displacement traces of untreated and treated sample of both triangular and square core structures of bamboo reinforced epoxy composites. Interestingly, treated core structures offered outstanding performance compared to the untreated systems. The composite structure treated using optimum conditions, i.e., 5.81 wt.%, a soaking time of 3.99 h, and a drying time of 72 h, exhibited the highest compressive strength. As can be seen from the force-displacement traces, the peak for this treatment condition was greater compared to untreated structures. This implied that the treatment was a good surface treatment for enhancing the interface bonding between bamboo fiber and the epoxy matrix [44], thus enabling the core structures to withstand higher the higher compressive forces exerted upon them.

**Figure 10:**
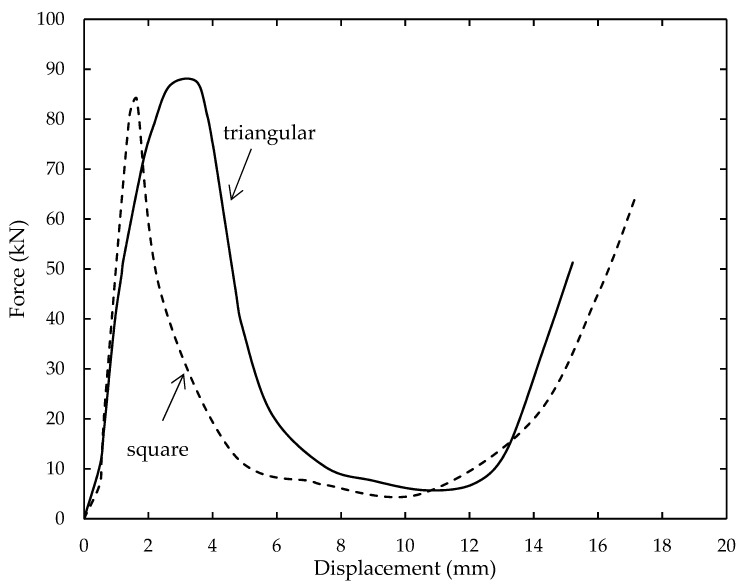
Typical force-displacement traces of square and triangular core structures.

In Figure 10, four typical phases could be observed with the elastic deformation region in the initial region. Here, the compressive load for square and triangular core structures steadily increases with displacement. Upon reaching the maximum load, the loading force exhibited by the triangular structure continues to increase slightly higher than square core. This could be explained by the differences in relative density of both cores where a core with higher relative density requires an immense amount of compressive load to deform. In addition, the relative density of the triangular structure was almost 20% of its square counterpart, thus explaining the higher peak loading experienced by the triangular core. Also, the prior cracks in the structures started to become visible as the stress was concentrated at this condition. In addition, the traces gradually decrease as the structures become unable to withstand the applied force. Thus, both cores started to fail. As the structures start to lose their rigid stability, the compressive load fell to almost half of the maximum compressive strength. Then, they reached a steady stage where the cell walls of the structures started to crack at their cell wall over almost half of the structure. The rupture of bamboo fibers in the composite was also noticeable. In addition, both structures show a failure mode of cracking at the cell wall thickness with a micro-buckling observed at the triangular core structure. The traces show that, after reaching the densification phase, both structures were utterly crushed, and the compressive load increased. Here, the overall compressive properties of triangular core structures outperformed the square counterparts for both untreated and treated samples. One factor that influenced the results is the core topology. According to Yamashita and Gotoh [45], a higher number of cells provide more support in resisting the compressive load. Their suggestion is in agreement with Hazizan and Cantwell [46], who reported that the same factor greatly influenced the portioning of energy. Moreover, the core with a smaller cell size is more preferable in the composite industry as this type of core is capable of preventing its structure from buckling under compressive load longer than a core structure with large cell size. Larger cells, unfortunately, tend to collapse inwards as the bonding between the fiber and matrix becomes weakened.

**Figure 11:**
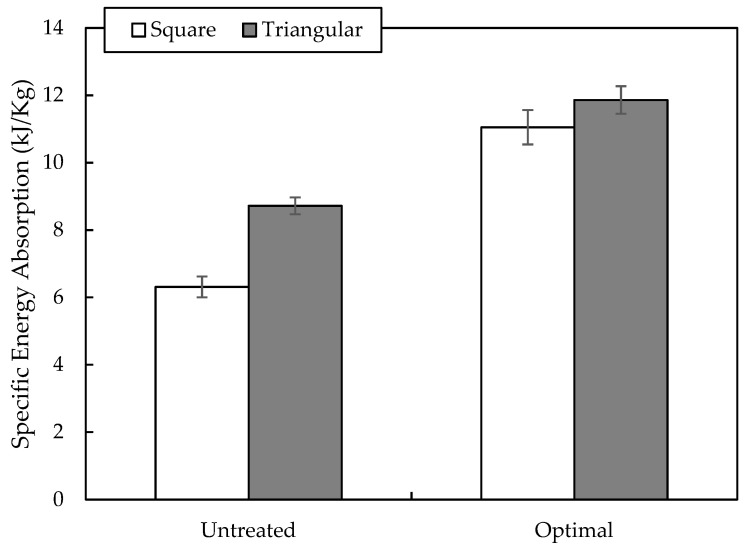
Energy absorption characteristics of bamboo reinforced epoxy square and triangular sandwich core structures.

Figure 11 shows the energy-absorbing characteristics for both square and triangular core structures. The specific energy absorption of the square and triangular composite core structures was calculated from the area under curve of force-displacement curves over the mass of the structures. The highest specific energy was recorded for the triangular core structure treated with the highest optimal NaOH concentration. On the other hand, the square structure with similar treatment conditions exhibited approximately 7% less specific energy than the triangular structure as the lower cell size and smaller number of cells render the square core incapable of withstanding greater compressive load compared to the triangular core. Furthermore, a triangular bamboo structure treated under optimal condition is highly superior than those treated composite by over 12%. Zuhri et al. [22] suggested that the energy-absorbing characteristics of the core structure can be affected by the poorer mechanical properties of the composite. In their study, they conducted compression tests on square and triangular flax/polypropylene and flax/PLA sandwich structures.

## 4. Conclusions

A series of mechanical tests were carried out on bamboo fiber subjected to a range of treatment conditions. The alkaline treatment conditions comprised NaOH concentration, soaking time, and drying time and were optimized using a Box–Behnken design. Then, BBD data were analyzed using ANOVA to produce the statistical results. It was found that the treatment variables were significantly involved in the mechanical properties of the bamboo reinforced epoxy composite. The optimum mercerization condition for bamboo is a NaOH concentration of 5.81 wt.%, a soaking time of 3.99 h, and a drying time of 72 h with a tensile strength of 338.95 MPa being recorded. The optimum alkaline treatment once again had a great effect on the structures where all the treated square and triangular cores outperformed the untreated fibers under compressive stress. The triangular structure based on treated bamboo reinforced epoxy composites shows an outstanding performance compared to its treated square counterpart in terms of compressive and specific strength. This is due to the high number of small cells. Again, the triangular core structure based on treated bamboo reinforced epoxy composite showed its superiority by absorbing 7% more energy compared to the treated square core structures.

## Figures and Tables

**Table 1:** The proposed variable values by the Box–Behnken Design-expert (6.0.11) software.

Independent Variables	Symbols	Coded and Actual Levels		
		Low (−1)	Middle (0)	High (+1)
Concentration of NaOH (wt.%)	X_1_	3	6	9
Soaking duration (hours)	X_2_	3	6	9
Drying duration (hours)	X_3_	24	48	72

**Table 2:** Experimental trials derived from Design-Expert Software.

RUN	Independent Variables					
	Coded Values			Actual Values		
	X_1_	X_2_	X_3_	X_1_	X_2_	X_3_
1	0	+1	−1	6	9	24
2	0	0	0	6	6	48 *
3	0	−1	−1	6	3	24
4	0	0	0	6	6	48 *
5	0	+1	+1	6	9	72
6	+1	−1	0	9	3	48
7	0	0	0	6	6	48 *
8	0	−1	+1	6	3	72
9	−1	+1	0	3	9	48
10	+1	0	+1	9	6	72
11	0	0	0	6	6	48 *
12	−1	−1	0	3	3	48
13	−1	0	+1	3	6	72
14	−1	0	−1	3	6	24
15	0	0	0	6	6	48 *
16	+1	+1	0	9	9	48
17	+1	0	−1	9	6	24

(* replication of middle level = 0:0:0).

**Table 3:** Alkaline treatment conditions and test results for bamboo reinforced epoxy composites.

No.	Conditions	Tensile Strength, *σ* (MPa)	Strain at Break, *ε* (%)	Young’s Modulus *E* (GPa)
0	Untreated bamboo fiber	138.88 ± 1.23	2.70 ± 0.45	4.96 ± 0.23
1	3 wt.% NaOH + 3 h soak + 48 h dry	276.98 ± 1.45	7.68 ± 3.30	3.05 ± 0.84
2	3 wt.% NaOH + 6 h soak + 24 h dry	253.09 ± 6.23	7.25 ± 1.33	3.66 ± 0.92
3	3 wt.% NaOH + 6 h soak + 72 h dry	312.29 ± 2.45	8.49 ± 2.47	3.14 ± 0.62
4	3 wt.% NaOH + 9 h soak + 48 h dry	268.82 ± 3.35	6.67 ± 1.78	3.74 ± 0.56
5	6 wt.% NaOH + 3 h soak + 24 h dry	263.74 ± 2.25	5.90 ± 2.13	3.78 ± 0.72
6	6 wt.% NaOH + 3 h soak + 72 h dry	339.27 ± 3.36	8.33 ± 2.73	3.70 ± 0.31
7	6 wt.% NaOH + 6 h soak + 48 h dry	296.19 ± 4.26	7.59 ± 1.55	3.25 ± 0.69
8	6 wt.% NaOH + 9 h soak + 24 h dry	301.52 ± 7.45	8.45 ± 3.34	3.00 ± 0.46
9	6 wt.% NaOH + 9 h soak + 72 h dry	296.19 ± 4.26	7.59 ± 1.55	3.25 ± 0.69
10	9 wt.% NaOH + 3 h soak + 48 h dry	260.13 ± 1.73	5.68 ± 2.05	4.50 ± 0.13
11	9 wt.% NaOH + 6 h soak + 24 h dry	226.85 ± 0.83	7.36 ± 0.86	3.43 ± 0.55
12	9 wt.% NaOH + 6 h soak + 72 h dry	304.92 ± 2.45	8.49 ± 2.47	3.14 ± 0.62
13	9 wt.% NaOH + 9 h soak + 48 h dry	242.25 ± 2.24	6.17 ± 1.06	4.39 ± 0.37

**Table 4:** Box–Behnken Design (BBD) analysis with experimental and predicted values of tensile strength.

Run	Independent Variables						Tensile Strength (MPa)			
	Coded Values			Actual Values			Experimental	Predicted	Residual	Error (%)
	X_1_	X_2_	X_3_	X_1_	X_2_	X_3_				
1	−1	0	+1	3	6	72	312.29	312.61	−0.325	−0.102
2	+1	0	−1	9	6	24	226.85	226.53	0.325	0.141
3	0	−1	−1	6	3	24	263.74	260.42	3.32	1.259
4	0	0	0	6	6	48	298.89	298.86	0.03	0.010
5	−1	−1	0	3	3	48	276.98	278.17	−1.19	−0.430
6	0	+1	+1	6	9	72	306.08	309.40	−3.32	−1.085
7	0	−1	+1	6	3	72	339.27	337.75	1.52	0.448
8	+1	−1	0	9	3	48	260.13	263.77	−3.64	−1.399
9	0	0	0	6	6	48	301.52	298.86	2.66	0.882
10	0	0	−1	6	9	24	251.55	253.07	−1.52	−0.604
11	0	0	0	6	6	48	297.25	298.86	−1.61	−0.542
12	+1	0	+1	9	6	72	304.92	302.79	2.13	0.699
13	−1	0	−1	3	6	24	253.09	255.22	−2.13	−0.842
14	0	0	0	6	6	48	300.45	298.86	1.59	0.529
15	0	0	0	6	6	48	296.19	298.86	−2.67	−0.901
16	+1	+1	0	9	9	48	242.25	241.06	1.19	0.491
17	−1	+1	0	3	9	48	268.82	265.18	3.64	1.354

**Table 5:** Statistical data from ANOVA for tensile strength.

Source	Response Value
R-Squared (*R*^2^)	0.994
Adjusted R-Squared	0.986
Predicted R-Squared	0.924
Standard Deviation	3.480
C.V. %	1.230
Adequate Precision	41.71
Mean	282.37

**Table 6:** ANOVA of the quadratic model for alkaline treatment of bamboo.

Source	Sum of Squares	df	Mean Square	F-Value	*p*-Value (Prob > F)
Model	14051.43	9	1561.27	129.17	<0.0001 *
X_1_- concentration	741.7	1	741.7	61.36	0.0001
X_2_- soaking	637.6	1	637.6	52.75	0.0002
X_3_- drying	8933.17	1	8933.17	739.08	<0.0001
X_1_^2^	23.62	1	23.62	1.95	0.2048
X_2_^2^	89.02	1	89.02	7.36	0.03
X_3_^2^	110.25	1	110.25	9.12	0.0194
X_1_ X_2_	2922.08	1	2922.08	241.76	<0.0001
X_1_ X_3_	461.67	1	461.67	38.2	0.0005
X_2_ X_3_	13.21	1	13.21	1.09	0.3306
Residual	84.61	7	12.09		
Lack of Fit	65.28	3	21.76	4.5	0.09 **
Pure Error	19.33	4	4.83		
Cor total	14136.03	16			

* Significant ** Not significant.
